# Somatic/gonadal mosaicism for structural autosomal rearrangements: female predominance among carriers of gonadal mosaicism for unbalanced rearrangements

**DOI:** 10.1186/s13039-015-0211-y

**Published:** 2016-01-28

**Authors:** Natalia V. Kovaleva, Philip D. Cotter

**Affiliations:** Department of Inherited Diseases, The Turner’s Scientific and Research Orthopaedic Institute for Children, Parkovaya Str. 64-68, St. Petersburg, 196603 Russian Federation; Department of Pediatrics, University of California San Francisco, San Francisco, CA 94143 USA; ResearchDx Inc., Irvine, CA 92618 USA

## Abstract

**Background:**

Mosaicism for chromosomal structural rearrangements (Rea) is rare and the timing and mechanisms of mosaic Rea formation, maintenance, and clinical manifestation are poorly understood. To date, there are no published data on the cytogenetic profile of mosaic Reas. The question as to whether the proportion of abnormal cells in the carrier’s cultured blood is clinically significant remains unanswered. A previous study showed a strong female preponderance among carriers of mosaicism for Rea with pericentromeric breaks, indicating female-specific instability in early embryos. However, there is no corresponding study on male to female sex ratio (SR) among carriers of somatic and/or gonadal mosaicism for non-centromeric Rea. Population rates of mosaic Rea carriers calculated from consecutive series of patients referred for various reasons and from prenatal samples have not been established. Therefore the objectives of the present study were several-fold: (1) a study on profiles of Rea involved, (2) comparative analysis of the proportion of cells with unbalanced Rea in blood cultures from asymptomatic and affected carriers, (3) comparative analysis of SR in carriers of mosaicism for balanced and unbalanced Rea, and (4) determination of the population frequency of mosaicism for autosomal Rea.

**Results:**

One hundred and three cases of mosaicism for autosomal non-centromeric Rea (N/Rea; normal line/structural rearrangement) in which the sex of the carrier had been specified were identified in the literature. Among balanced Rea, there was a prevalence of reciprocal translocations (89 %) over inversions (11 %). Among unbalanced Rea, deletions were the most frequent (40 %), followed by duplications (25 %) and rings (16 %). Derivatives and other chromosome abnormalities were less frequent (9 and 10 %). Eight of eleven (73 %) affected carriers of unbalanced Rea displayed a high proportion (>50 %) of abnormal cells compared to 4/37 (11 %) in asymptomatic carriers, *p* < 0.0001. Among carriers of mosaicism for balanced Rea there was a slight male predominance, 24 M/22 F, unlike the strong female predominance among carriers of mosaicism for unbalanced Rea, 11 M/46 F, *p* < 0.0001. Among ten carriers of unbalanced Rea with reproductive failure, only one was a male with infertility, and one was a partner of a woman experiencing recurrent spontaneous abortion. Population rates of mosaics for reciprocal translocaton (N/rcp), inversion (N/inv), and unbalanced Rea (N/unbal Rea) calculated from published data on consecutive series of patients with reproductive failures were 0.02 ‰, 0.005 ‰, and 0.002 ‰, correspondingly. Among 30,376 infertile patients three carriers of mosaicism for balanced Rea were identified (two cases of N/rcp and one case of N/inv), whereas among 26,384 patients with habitual abortion seven carriers were detected (five N/rcp and two N/inv). Among all 56,760 tested patients with reproductive failures only one was found to be a carrier of mosaicism for an unbalanced Rea (N/del, mosaicism for deletion).

**Conclusions:**

A high proportion of Rea cells (>50 %) detected in cultured T-lymphocytes is associated with clinical manifestation of chromosomal imbalance. A strong female prevalence among carriers of mosaicism for unbalanced Rea suggests male-specific selection against abnormal cells rather than impairment of male gametogenesis, as the latter suggests a better prognosis for male fetuses. These findings should be taken into consideration when genetic counseling of patients referred after a diagnosis of mosaicism for an unbalanced rearrangement in a fetus.

## Background

Mosaicism for structural chromosome abnormalities is rare and may be challenging for genetic counseling, particularly when detected prenatally. The identification and counseling of gonadal mosaicism (GM) may be even more problematic, being both asymptomatic and cryptic in the GM carrier.

There are two hypotheses for the existence of GM discussed in the literature. One is that the mutation occurs in a germ cell that continues to divide (mosaicism confined to germ cells). The other possibility is that the mutation occurs very early in a somatic cell before the separation to germinal cells and is therefore present both in somatic and germinal cells. Depending on various factors, such as the gene(s) involved and/or the degree of mosaicism, the carrier of a somatic and/or germline mosaicism may be asymptomatic (making GM difficult to detect) or may present with various symptoms of the disease [1]. A previous study reported a strong female preponderance among carriers of mosaicism for Rea with pericentromeric breaks indicating female-specific instability in early human embryos [2]. No data is available on the sex ratio among carriers of GM for non centromeric Rea.

The question as to whether the proportion of Rea cells in the carrier’s cultured blood is clinically significant is unresolved. Data from Cheung et al. [3] confirmed the previous suggestion of Pagon et al. [4] that chromosome analysis of stimulated T-lymphocytes does not reflect the true rate of abnormal cells in a carrier of mosaicism. Considering that blood cultures (i.e. stimulated T-lymphocytes) are commonly employed in routine cytogenetic examination, it is of importance to study the problem thoroughly.

The objectives for this study were: (1) a study on profiles of Rea involved, (2) comparative analysis of the proportion of cells with unbalanced Rea in blood cultures from asymptomatic and affected carriers, (3) comparative analysis of SR in carriers of mosaicism for balanced and unbalanced Reas, and (4) determination of the population frequency of mosaicism for autosomal Rea.

## Materials and methods

We reviewed reports in the literature of mosaicism for N/Rea cases detectable microscopically (up to 850-band level of resolution) either by conventional cytogenetics or by molecular cytogenetics. The cases were identified from various sources including PubMed using combinations of the search terms “mosaicism”, “mosaic”, ”recurrent”, “inherited”, “familial”, ‘transmitted”, “maternal’, “paternal”, and “parental”. Only reports of N/Rea carriers of known sex were selected for the study. From the sample collected we excluded cases of Rea with both breakpoints localized at pericentromeric regions, because of the strong female preponderance among carriers of such mosaicism [2, 5]. Cases of Rea transmitted from a carrier parent rescued along with the formation a normal line were similarily not included in the study. The majority of the cases reported since 2000 were detected, verified and/or analysed using molecular cytogenetic and molecular technologies.

One hundred and four cases of carriers of N/Rea, along with the data on their chromosome constitution, carrier’s age at the birth of the proband (when relevant and/or specified), proportion of abnormal cell line(s), and the indication for testing have been identified and subdivided as follows: affected carriers of apparent GM with abnormal offspring, asymptomatic carriers of GM with abnormal offspring, asymptomatic carriers of GM with healthy offspring, asymptomatic carriers of somatic mosaicism (SM) assumed to have GM, i.e. patients with poor reproductive history, and asymptomatic carriers of somatic mosaicism fortuitously detected. According to Barber [6] (the majority of the cases of affected carriers with abnormal offspring in the present study were retrieved from this review), individuals were considered phenotypically affected when any type of phenotypic anomaly was reported even if the etiological role of the chromosome abnormality in the same individual is questionable. We also analyzed available published data on consecutive series of patients/couples experiencing reproductive problems, aiming to estimate a population frequency of N/Rea mosaicism. Data were analyzed using standard statistics, a Chi-square test with Yates correction. The comparison of observed and expected proportions was made using binomial test.

## Results and discussion

### N/Rea profile in studied groups

There is a prominent difference between studied groups regarding the proportion and profiles of unbalanced and balanced Rea. As seen from Table 1, among 12 affected N/Rea carriers with affected offspring who inherited the same non mosaic Rea, there were no carriers of balanced translocations. Among them, carriers of deletions (including ring chromosomes) and duplications were represented equally.

Among asymptomatic carriers with affected offspring (Table 2), 33 of 42 were mosaics for an unbalanced Rea, with some prevalence of deletions (16 cases including ring chromosomes) over duplications (8 cases). Additionally, there were four cases of unbalanced translocation and five cases of other Rea. Among mosaics for balanced Rea, two were carriers of an inversion, six were carriers of a reciprocal translocation, and one was a carrier of insertion.

**Table 1:** Saawomatic/gonadal mosaicism for non centromeric rearrangement in affected carriers with affected offspring

Reference	Karyotype	Age at birth of the proband	Proportion of abnormal cell line(s)	Indication for testing
*Unbalanced rearrangements*				
*N/del*				
Freitas et al., 2012 [25]	46,XX/46,XX,del(2)(q36.1q36.3)	23 yr	90% BL, 10% DNA	mild presentation of MFDH; a child with the same Rea
Naritomi, Hirayama, 1989 [26]	46,XX/46,XX,del(8)(q23.3q24.13)	34 yr	50%	mild trichorhoniphalangeal syndrome I in the mother, a child with the same Rea
Magenis et al., 1989 [27]	46,XX/46,XX,del(9q31.3)	ns	ns	mild mental retardation, affected child with the same Rea
Zori et al., 1993 [28] (patient B)	46,XX/46,XX,del(17)(p11.2p12)	30 yr	55% BL	partially affected, a child with SMS
*N/dup*				
Cox et al., 2002 [29]	46,XX/46,XX,dup(7)(?p15.3?p22)	40 yr	83% BL	global intellectual impairment, rebellious behavior, craniofacial dysmorphism, a child with the same Rea
Kennedy et al., 2001 [30]	46,XY/46,XY, dup(8)(p23.1p23.1)	ns	68% BL	congenital heart defect, a child with the same Rea
Pfeiffer and Schutz, 1993 [31]	46,XX/46,XX,dir dup(11)(q23->qter)	26 yr	19% BL	mildly retarded; a dysmorphic child with the same Rea
Barber et al., 2006 [32] (family 1)	46,XX/46,XX, inv dup ins(16)(q11.2q13q11.2)	33 yr	52% BL	developmental delay and phenotypic abnormality, affected child with the same Rea
Moog et al., 1994 [33] (patient B)	46,XX/46,XX,dup18(pter->cen)	26 yr	80% BL	dysmorphic, slightly mentally retarded, son with dup(8)
*N/ring*				
Fryns, Van den Berghe, 1979 [34]	46,XX/46,XX,r(22)/45,XX,t(15q21q)	21 yr	32%/65% BL, 19%/24% SF	slightly mentally retarded, a child with the same Rea
*N/t unbalanced*				
De Pater et al., 2003 [35]	46,XX,der(18)t(18;21)(q21.3;p12)/46,XX,der(21)t(18;21)(q21.3;p12) ^a^	37 yr	47%/53% BL	very mild phenotypic abnormalities, a child with 18q- syndrome
*N/other rea*				
Galjaard et al., 2003 [36]	46,XY/46,XY, t(4;7)(p15.2;q35), microdeletions at both der(4) and der(7)	ns	70% BL, 96% SF	isolated postaxial polydactyly, affected child with the same Rea
Total	2 males 10 females			

^a^ presence of normal cell line can be suggested confidently because of very mild clinical manifestation; healthy 46,XY child

BL, blood culture (i.e. stimulated T-lymphocytes)

SF, skin fibroblasts culture

**Table 2:** Somatic/gonadal mosaicism for non centromeric rearrangement in asymptomatic carriers with affected offspring

Reference	Karyotype		Carrier's age at birth of the proband	Proportion of abnormal cell line(s)	Indication for testing
Unbalanced rearrangements					
N/del					
Galan-Gomez et al., 1994 [37]		46,XX/46,XX,del(5)(p14-pter)	ns	ns	children with 5p- syndrome
Johnson et al., 2000 [38]		46,XY/46,XY,del(5)(p14)	ns	100% BL, 99% SF	affected child with the same deletion
McDonald et al., 1988 [39] (family 3)		46,XX/46,XX,del(5)(p14)	ns	ns	a child with del(5)
Niebuhr, 1978 [40]		46,XX/46,XX,del(5)(p14)	ns	5% BL, 3% SF	a child and fetus with the same Rea
Van Tuinen et al., 2001 [41]		46,XX/46,XX,del(5)(p14.2)	ns	4% BL	a child with cri-du-chat syndrome
Brandriff et al., 1988 [42]		46, XX/46,XX,del(13)(q22q32)	ns	0% BL, 0% SF a)	a chid and fetus with the same Rea
Michalova et al., 1982 [43]		46,XX/46,XX,del(13)(q12->q31)	18 yr	3% BL	child with retinoblastoma
Kokkonen, Leisti, 2000 [44]		46,XX/46,XX,del(15)(q11q13)	ns	0% BL b)	two affected children
Rump et al., 2008 [45]		46,XX/46,XX,del(15)(q26.2->qter)	ns	0% BL b)	two children with del(15)
Sanchez et al., 2014 [46]		46,XX/46,XX, del(15)(q11.2q13)	ns	0% BL, 0% normal SF, 35% hypopigmented SF	dyzygotic twins with Angelman syndrome
Hoo et al., 1985 [47]		46,XX/46,XX,del(16)(q11.1q12.1)	22 yr	0% BL, 0% SF c)	two children with del(16)
Garcia-Heras et al., 2005 [48]		46,XX/46,XX,del(20)(p11.1p12)	33 yr	25% BL	child with the same deletion
N/dup					
Eussen et al., 2007 [49]		46,XX/46,XX,inv dup(2)(q34q33)	28 yr	19% BL	two children with the same Rea
Bernardini et al., 2005 [50]		46,XX/46,XX,dup(4)(p15p15)	young	30% BL, 20-25% different tissues	three abortions with the same Rea
Toska et al., 2010 [51]		46,XX/46,XX,dup(4)(q22.2q23)	24 yr	0% BL b)*	two siblings with dup(4)
Fan et al., 2001 [52] (family 2)		46,XY/46,XY,dup(8)(p21.3p23.1)	ns	20% BL	two children with the same Rea
Tonk et al., 1996 [53]		46,XX/46,XX,dir dup(10)(q24.2->q24.3)	ns	10%	two children with dup(10)
Hocking et al., 1999 [54]		46,XY/46,XY,dup(13)(q32q34)	ns	ns	a child with the same Rea
Babovic-Vuksanovic et al., 1998 [55]		46,XX/46,XX,dup(17)(q24q25.1)	16 yr	29% BL	recurrent abortins, two children with dup(17)
Flowers et al., 2015 [56]		46,XX/46,XX,dup(18)(q12.1q21.1)	38 yr	20% BL	a fetus with the same Rea
N/ring					
Meza-Espinoza et al., 2008 [57]		46,XY/46,XY, r(17)	ns	4% BL	a child with multiple anomalies with the same Rea
Fryns et al., 1992 [58]		46,XX/46,XX,r(18)(p11.3q23)	26 yr	8% BL	polymalformed child with r(18)
Flejter et al., 1996 [59]		46,XX/46,XX,r(19)	27 yr	4% BL	affected child with the same Rea
Phelan, 2008 [60]		46,XX/46,XX,r(22)	ns	ns	affected child with the same Rea
N/t unbalanced					
Engel et al., 2001 [61]		46,XX/46,XX,psudic(5;21)(q12.p13)	young	0% BL, 0% SF b)	two children with psudic (5;21)
Kouru et al., 2011 [62]		46,XX/45,XX,psu dic(5;22)(p15.p11.1)	ns	0% BL, 5% SF	a child and a fetus with the same Rea
Gijsbers et al., 2011 [63] (case 2)		46,XX/46,XX,der(22)t(8;22)(q24.2;p10)	ns	52% BL	affected daughter with the same Rea
Papenhausen et al., 1991 [64]		46,XX/46,XX,der(21)t(21;21;)(p11;q22.1)	29 yr	30% BL	a child with the same Rea
N/other rea					
Al Arrayed, 1998 [65] (case 10)		46,XY/46,XY,multiple rea(2)	ns	ns	three abnormal children
Eckel et al., 2006 [66]		46,XX/46,XX,trp(12)(pter->p11.22->p12.3::p12.3->qter)	ns	12% BL	affected child with the same Rea
Masada et al., 1989 [67]		46,XY/46,XY, del(14)(q32.11->qter)/46,XY,dup(14)(q32.11->qter)	ns (mother 31 yr)	0% BL ,0% SF d)	a child with del(14), a child with dup(14)
Insley et al., 1968 [68]		46,XX/46,XX,Dq+	22 yr	2% BL, 3% SF	two daughters with the same Rea
D'Angelo et al., 2010 [69]		46,XX/46,XX,del(20)(p11.21)dup(20)(p11.21.p13)	23 yr	15% BL	affected daughter with the same Rea
Total		6 males 27 females			
Balanced rearrangements					
N/inv					
Shapira et al., 1997 [22]	46,XY/46,XY,inv(9)(p24q34.1)		25 yr	25% BL	a child with recombinant 9p aneusomy
Wang et al., 2010 [70]	46,XY/46,XY,inv(20)(p12.2q13.33)		35 yr	50% BL	two children with recombinant chromosome 20
N/t balanced					
Aurias et al., 1978 [71]	46,XX/46/XX,t(2;4)(q37;q28)		ns	ns	a child with der(4) t(2;4)(q37;q28)
Becker, Albert, 1963 [72]	46,XY/45,XY,nonacrocentric t(2;21)		23	55% BL	neurofibromatosis, a child with Down syndrome
Gardner et al., 1994 [73] (case 7)	46,XX/46,XX,rcp(5;18)(p15;q21)		ns	0,1% BL, 0% SF	a child with der (18)
Simonova et al., 2005 [74]	46,XY/46,XY,t(5;20)(p12;q13)		ns	8% BL	a child with del(5)
Sciorra et al., 1992 [75]	46,XY/46,XY,t(7;14)(q36;q1?)		ns (mother 31 yr)	0.5% BL, 0% SF	a child with 7q+
Opheim et al., 1995 [76] (case 2)	46,XX/46,XX,t(8;13)(p23.2;q21.2)		ns	57% BL	a child with der(8)t(8;13)
Yatsenko et al., 2009 [77]	46,XX/46,XX,ins(12)(q12p11.1p13.1)		ns	50% BL	two children with Noonan syndrome and the same Rea
Total	5 males 4 females				

a) ovarian germinal mosaicism deduced from absence of the Rea in sperm chromosomes

b) ovarian germinal mosaicism deduced from molecular analysis

c) maternal origin proved by 16qh heteromorphism

d) paternal origin proved by 14p heteromorphism

A different proportion between unbalanced and balanced Rea was observed in the remaining three groups of patients with SM/GM. Asymptomatic carriers with healthy offspring carrying the same Rea (Table 3), showed a substantial prevalence of balanced Rea (8 of 10 cases). In the group of asymptomatic carriers with poor reproduction (Table 4) balanced Rea also prevailed over unbalanced Rea (23 cases vs 10 cases). Among the latter group there was a significant predominance of deletions including rings over duplications (9 cases vs 1 case). Finally, among asymptomatic carriers of SM detected fortuitously (Table 5), we detected six carriers of a balanced Rea.

**Table 3:** Somatic/gonadal mosaicism for non centromeric rearrangement in asymptomatic carriers with unaffected offspring

Reference	Karyotype	Age at ascertainment	Proportion of abnormal cell line(s)	Indication for testing
*Unbalanced rearrangements*				
Tinkel-Vernon et al., 2001 [78]	46,XY/46,XY,del(21)(q11.2->q21)	ns	50% BL	normal son with the same Rea
Mazzaschi et al., 2011 [79]	46,XX/46,XX,r(21)	40 yr	30% BL	normal child with the same Rea
Total	1 male 1 female			
*Balanced rearrangements*				
Kleczkowska et al., 1990 [21] (case 1)	46,XY/46,XY,t(1;9)(p13.1;p12.2)	ns	50% BL	Two healthy children the same Rea
Zackovsi et al., 1995 [80]	46,XY/46,XY,inv(1)(p31.2p34.3)	ns	28% BL	normal child with inv(1)
Heil et al., 1997 [81] (case 1)	46,XY/46,XY,t(2;3)(q37;q21)	ns	19% BL	a fetus with t(2;3)
Farrell, 1991 [82] (case 2)	46,XX/46,XX,rcp(5;18)(q35;q21.3)	ns	28% BL	46,XY, t(5;18) son with reproduction failure
Leegte et al., 1998 [83] (case 1)	46,XX/46,XX,t(9;15)(q12;p11.2)	ns	32% BL	46,XY, t(9;15) son with infertility
Dupont et al., 2008 [84]	46,XX/46,XX,t(9;22)(q34.3;q13.3)	ns	50% BL	affected grandchild with 46,XY,der(22)t(9;22) (q34.3;q13.3)
Storto et al., 1999 [85]	46,XY/46,XY,10qs	ns	60% BL	normal child with 10qs
Gardner et al., 1994 [73] (case 12)	46,XX/46,XX,rcp(11;22)(q23;q21)	ns	62% BL	a grandchild with der(22)t(11;22)
Total	4 males 4 females			

x

**Table 4:** Somatic mosaicism for non centromeric rearrangement in asymptomatic carriers with poor reproductive history

Reference	Karyotype	Age at ascertainment	Proportion of abnormal cell line(s)	Indication for testing
*Unbalanced rearrangements*				
*N/del*				
D'Alessandro et al., 1992 [86]	46,XX/46,XX,del(6)(p23)	ns	11% BL	recurrent abortions and idiopathic hyporpolactinemia
Reddy, 1999 (case 3) [87]	46,XX/46,XX,del(8)(p23.1)	29 yr	22% BL	recurrent SA
Kleczkowska, Fryns, 1990 [88]	46,XX/46,XX,del(11)(:q14.2->q23.2:)	28 yr	25% BL	recurrent SA
Dutta et al., 2011 [89]	46,XX/46,XX,del(17)(q)	ns	ns	recurrent miscarriage
Sachs et al., 1985 [90]	46,XX/46,XX,del(20)(p12)	ns	22%	recurrent SA
*N/dup*				
Somprasit et al. 2004 [91]	46,XY/46,XY,dup(21q22.13-q22.2)	33 yr	0% BL, 7% sperm	recurrent SA
*N/ring*				
Lee, 2002 [92]	46,XX/46,XX,r(4)/45,XX,-4/46,XX,dic r(4)/47,XX,r(4),+r(4)	27 yr	75%/8%/5%/4%	infertility and short stature
Scholtes et al., 1998 [93]	46,XX/46,XX,r(14)	ns	ns	infertility, pre-ICSI testing
Tarlatzis et al., 2000 [94]	46,XX/46,XX,r(14)	ns	ns	infertility
Hammoud et al., 2009 [95]	46,XY/46,XY,r(21)/45,XY, −21	ns	95%/3% BL, 7%/0% sperm	infertility, pre-ICSI testing
Total	2 males 8 females			
*Balanced rearrangements*				
*N/inv*				
Gekas et al., 2001 [96]	46,XY/46,XY,inv(10)(p11q21)	ns	39%	sterility, candidate for ICSI
Kleszkowska et al., 1990 [21] (case 4)	46,XX/46,XX,inv(12)(q12q24)	25 yr	90%	two SA
De la Fuente-Cortes et al., 2009 [15]	46,XY/46,XY,inv(14q)	ns	6% BL	repeated miscarriages
*N/t balanced*				
Stenchever et al., 1977 [97]	46,XX/46,XX.t(1;2)	27 yr	50% BL	habitual abortion
Stenchever et al., 1977 [97]	46,XX/46,XX,t(1;16)	23 yr	50% BL	habitual abortion
Northup et al., 2007 [98]	46,XX/46,XX,psu dic(1;19)(q10;q13.42)	27 yr	10% BL, 0% SF	premature ovarian failure
De la Fuente-Cortes et al., 2009 [15]	46,XX/46,XX, t(1p;21q)	ns	2% BL	repeated miscarriage
Almeida et al., 2012 [99]	46,XY/46,XY,t(2;2)(p23;q21.2)	31 yr	100% BL, 84% sperm	infertility, one SA
Lebbar et al., 2008 [100] (patient 2)	46,XY/46,XY,t(2;4;12)	40 yr	30% BL	secondary infertility, severe oligoasthenospermia, necrospermia, leucospermia, teratospermia, one child (not tested)
Stenchever et al., 1977 [97]	46,XX/46,XX,t(2;7)	19 yr	75%	habitual abortion
Stenchever et al., 1977 [97]	46,XX/46,XX,t(2;8)	24 yr	50%	habitual abortion
Shaham et al., 1992 [101]	46,XX/46,XX,t(2;16)(p23;q24)	ns	ns	recurrent SA
Cantu and Ruiz, 1986 [102]	46,XY/46,XY, t(3;4)(q22;q35)	ns	ns	recurrent SA
Farrell, 1991 [82] (case 1)	46,XX/46,XX,t(3;6)(q13.2;q25.3)	ns	9%	recurrent SA
Sciorra et al., 1985 [103]	46,XX/46,XX,t(4;5)(4pter->4q21::5q32->5qter;5pter->5q34::4q21->4qter)	ns	17-24% BL, 0% SF	infertility, one miscarriage
De la Fuente-Cortes et al., 2009 [15]	46,XY/46,XY,t(4q;9q)	ns	2% BL	recurrent SA
Meza-Espinoza et al., 2008 [57]	46,XY/46,XY,t(5;16;17)	ns	16% BL	habitual abortion
Kleszkowska et al., 1990 [21] (case 2)	46,XY/46,XY,rcp(9;13)(p21;q13)	29 yr	70%	repeated miscarriage
Tuerlings et al., 1998 [104]	46,XY/46,XY,t(9;20)(p22;p13)	ns	ns	two SA at 1st trimester
Lebbar et al., 2008 [100] (patient 1)	46,XY/46,XY,t(12;14;12;9)(q13;q32;p13;q32)	52 yr	20% BL	secondary infertility, variable moderate oligospermia, two healthy children
Gekas et al., 2001] [96]	46,XY/46,XY,t(10;13)(p13.2;q21)	ns	19% BL	sterility, candidate for ICSI
Gekas et al., 2001 [96]	46,XY/46/XY,t(11;19)(p11.2;q12)	ns	79% BL	sterility, candidate for ICSI
Clementini et al., 2005 [105]	46,XY,/46,XY,t(15;20)	ns	3% BL	sterility, candidate for ICSI
Total	13 males 10 females			

Overall, among balanced Rea, there was a prevalence of reciprocal translocations over inversions (89 and 11 %). As to the distribution of unbalanced Rea, deletions were the most frequent (40 %), followed by duplications (24 %), and rings (17 %). Derivatives and other chromosome abnormalities were less frequent (9 and 10 %).

**Table 5:** Somatic mosaicism for non centromeric rearrangementin asymptomatic carriers, fortitous findings

Reference	Karyotype	Age at ascertainment	Proportion of abnormal cell line(s)	Indication for testing
Balanced rearrangements				
Kleszkowska et al., 1990 [21] (case 1)	46,XY/46,XY,rcp(1;9)(p13.1;p12.2)	27 yr	50% BL	fortitous finding
Schmid, Hatfield, 1962 [106]	46,XX/46,XX, tan(2;13,14,or15)(p11.2; q26,32,or34)	86 yr	25% BL	a child and a grandchild with a different Rea
Leegte et al., 1998 [83]	46,XX/46,XX, t(3;7)(q26.2;p14)	64 yo	40 % BL	46,XY son with two stillborn children
de Pina Neto, Ferrari, 1980 [107]	46,XX/46,XX,t(3;20) de novo	6 yr	54% BL	a sibs with a different maternal Rea
Couzin et al., 1987 [108]	46,XY/46,XY,t(7;14)(q32;q11)	adult	8% BL	a child with trisomy 21
Kleszkowska et al., 1990 [21] (case 3)	46,XX/46,XX,ins(14;13)(q24.1;q31.1q32.3	24 yr	60% BL	trilogy of Fallot, 46,XY child with tetralogy Fallot
Total	2 males 4 females			

A low proportion of mosaics for derivative chromosome can readily be explained by the mechanism of their formation, i.e. postzygotic non-homologous recombination or nonhomologous end-joining [7].

#### Proportion of cells with unbalanced Rea in blood cultures from asymptomatic and affected carriers

The proportion of abnormal cells was reported in 89 cases. On average, in asymptomatic carriers of a balanced Rea (*n* = 41), the mean proportion of abnormal cells was 33 %, and the corresponding figure for asymptomatic carriers of unbalanced Rea (*n* = 38) was 20 %. In contrast, the mean proportion of abnormal cells in affected carriers of unbalanced Rea (*n* = 11) was 63 %. Since the number of tested cells was not specified in every case, a valid statistical analysis of the figures obtained was not possible.

Therefore, we analyzed a number of individuals with a proportion of abnormal cells reported to be larger than 50 %. A remarkable difference was found between asymptomatic carriers of unbalanced Rea and affected carriers of unbalanced Rea: 4 of 37 (11 %) vs 8 of 11 (73 %), *p* < 0.0001. Unfortunately because of few reports of such cases, the size of the latter group is small.

The reliability of routine chromosome analysis of stimulated T-lymphocytes from blood for detection and evaluation of mosaicism was questioned when higher rates of detection of mosaicism in cultured skin fibroblasts became evident [8–10]. Recent studies using array CGH confirmed that conventional cytogenetic methods underestimate the level of mosaicism [3]. However, although undoubtedly array CGH and single-nucleotide polymorphism (SNP) microarrays are superior to other methodologies in detecting somatic chromosome mosaicism [3, 11], it should be acknowledged that currently conventional chromosome analysis is the most readily available method worldwide and will be so in the foreseeable future. Therefore, awareness of an association of a high rate of abnormal cells in cultured T-lymphocytes with clinical manifestation of chromosomal imbalance might be helpful, particularly if this is confirmed in studies on prenatal cases.

Nevertheless, it should be noted that when GM is suspected in the absence of SM in blood cultures, further application of modern technologies is desirable, either for searching for the abnormal cell line(s) in different tissues or for identification of the parental origin of the recurrent Rea detected in the offspring.

#### Sex ratio in carriers of GM for balanced and unbalanced Reas

As summarized in Table 6, among affected carriers of GM, there is a notable female predominance (2 M/10 F). Among asymptomatic carriers of unbalanced Rea, both carriers of GM and carriers of SM, there is also a significant prevalence of females (9 M/38 F and 2 M/8 F). In contrast, both asymptomatic carriers of proven GM and asymptomatic carriers of SM for balanced Rea show a slight, but not significant, prevalence of males (9 M/8 F and 15 M/14 F, correspondingly). Overall, carriers of unbalanced Rea demonstrate a highly significant fiour-fold female predominance (11 M/46 F, SR = 0.24), different from population ratio of 1.06 at *p* < 0.0001, while male predominance (SR = 1.09) among carriers of mosaicism for balanced Rea is not different from population ratio of 1.06.

**Table 6:** Sex ratio in carriers of somatic/gonadal mosaicism for structural and autosomal mosaicism

Group	Unbalanced rearrangements		Balanced rearrangements	
	Males	Females	Males	Females
Affected carriers of gonadal mosaicism	2	10		
Asymptomatic carriers of gonadal mosaicism, abnormal offspring	6	27	5	4
Asymptomatic carriers of gonadal mosaicism, unaffected offspring	1	1	4	4
Subtotal, n=64	9	38	9	8
Asymptomatic carriers of somatic mosaicism, poor reproductive history	2	8	13	10
Asymptomatic carriers of somatic mosaicism, fortotous findings			2	4
Subtotal, n=39	2	8	15	14
Total, n=103	11	46	24	22
Sex ratio	0.24 *		1.09	

* different from population ratio of 1.06, p < 0.0001

Considerable, but not several-fold, prevalence of females over males among carriers of non-mosaic reciprocal translocations, both referred for prenatal testing for the presence of chromosomal Rea and those diagnosed as Rea carriers during prenatal testing, is well documented [12–14]. A similar female predominance was found among carriers of reciprocal translocations experiencing repeated miscarriages (see Table 7). This has been commonly explained by male sterility [15–18]. However analysis of the literature (Table 7) did not show a correspondingly significant predominance of males over female among infertile carriers of reciprocal translocation (SR = 1.2, not significantly different from 1.06). Moreover, the rate of reciprocal translocations in infertile males is even lower compared to the reciprocal translocations rate in males from couples experiencing repeated miscarriage (0.53 % vs 0.86 %).

**Table 7:** Autosome rearrangements in patients with reproductive failure

Groups	No. of carriers of non mosaic rearrangement		Balanced nonmosaic rearrangements, n (%)			Other non mosaic rearrangements	Mosaicism for structural rearrangement excluding SCM		Source
			Reciprocal translocation	Robertsonian translocation or isochromosome	Inversion		Non centromeric rearrangement	Centromeric rearrangement	
Couples with infertility (n=27,168)	Males (n=13,573)	156	63	70	20	1		2 (rob)	[93, 105, 109–117]
	Females (n=13,595)	111	51	22	36	2			
	Sex ratio		1.2	3.8	0.6				
Patients with infertility (n=3,208)	Males (n=2,196)	44	20	18	3	1	3 (2 rcp, 1 inv)		[96]
	Females (n=1,012)	21	7	7	7				
Patients and couples with infertility, total (n=30,376)	Males (n=15,769)	196 (1.3%)	83 (0.53 %)	88 (0.56%)	23 (0.15%)	2	3 (2 rcp, 1 inv)	2 (rob)	
	Females (n=14,607)	132 (0.9%)	58 (0.4%)	29 (0.2%)	43 (0.29%)	2			
Couples with repeated miscarriages (n=26,384)	Males (n=13,192)	187 (1.5%)	113 (0.86%)	54 (0.41%)	19 (0.14%)	1	5 ( 4 rcp, 1 inv)		[15, 18, 57, 89, 105, 117–126]
	Females (n=13,192)	318 (2.5%)	203 (1.54%)	94 (0.74%)	18 (0.14%)	3	3 (1 rcp, 1 inv, 1 del)		
	Sex ratio		0.56	0.56	1.1				
Combined data	56,760	833	439	262	101	8	11 (7 rcp, 3 inv, 1 del)	2 (rob)	

While one might expect a female predominance among asymptomatic carriers of GM for balanced Rea (mostly reciprocal translocations), who were diagnosed as such because of their abnormal offspring, this was not observed. However, among carriers of GM for unbalanced Rea there was a strong female prevalence. The same profile was found in the subgroup of carriers of SM mosaicism.

Mosaicism for unbalanced Rea does not appear to be a significant reason for male sterility, since among ten asymptomatic carriers with reproductive failure (Table 4) only one was a male with infertility, and another was a partner of a woman experiencing recurrent spontaneous abortion. Moreover, as seen from Table 7, among 200 infertile males diagnosed as carriers of a chromosome abnormality, none were diagnosed as a carrier of SM for unbalanced Rea. Consequently, other mechanism(s) resulting in the strong female predominance among carriers of mosaicism for unbalanced Rea can be postulated, including a high intrauterine lethality of male carriers, a male-specific selection against abnormal cells in the early embryo development, or a high instability in the early female embryo development.

A high intrauterine lethality of male carriers can be excluded because of significant predominance of females among abortuses with mosaicism for unbalanced Rea (Kovaleva, unpublished). Male-specific selection against abnormal cells in early embryo development seems more plausible. Several authors suggested that female embryos are relatively delayed in early embryonic development [19, 20]. The delay in early female development has been ascribed to the absence of a Y chromosome. However, the process of X inactivation, since it may occur when there are ≤ 10 cells in the embryo might itself contribute to a slight delay in early female embryo development [19]. A higher male cell turnover might facilitate effective selection against abnormal cell line.

High instability in early female embryo development would predict a female prevalence would be expected among both carriers of balanced and unbalanced Rea, arguing against this mechanism. However, the strong female prevalence is only observed among carriers of unbalanced Rea. Additional studies of the phenomenon of multifold female predominance among carriers of somatic and/or gonadal mosaicism for unbalanced Rea will add a new dimension to diversity of manifestation of human sexual dimorphism.

#### Estimation of detection frequency of somatic N/Rea mosaicism

The results of the combined data on structural autosomal Reas excluding supernumerary marker chromosomes (SCM) detected in 56,760 patients referred for chromosome testing for reproductive failure are presented in Table VII. Among them, 833(1.46 %) individuals were found to be carriers of structural chromosomal non mosaic abnormalities, and 13 (0.02 %) were carriers of N/Rea. Among balanced Reas, mosaics for inversions were the most frequent (3/103 = 2.9 %), the reciprocal translocations (7/453 = 1.5 %), while mosaics for Robertsonian translocation were less frequent (2/265 = 0.8 %), A majority of mosaics (10/11) were balanced Reas, and only one of 56,760 tested patients with reproductive failure was a carrier of mosaicism for unbalanced Rea.

The data on the incidence of mosaicism for balanced Reas obtained from the analysis of studies on patients with reproductive failures are consistent with corresponding data from a report on a constitutional chromosome analysis in 74,306 consecutive patients [21]. They reported an incidence of N/rcp carriers among all reciprocal translocation carriers as 1: 120 and incidence of N/inv carriers among all inversion carriers as 1 : 25. Corresponding figures from the present study are 1: 65 and 1: 34.

It was noted above that two groups of patients with reproductive problems, i.e. patients with infertility and patients with repeated miscarriage, differ by both rate of chromosome abnormalities and SR among carriers of chromosome abnormalities. In couples with repeated miscarriage there was a notable female predominance among carriers of reciprocal translocations (113 M/203 F, SR = 0.56) unlike a slight male predominance among infertile couples (63 M/51 F, SR = 1.2). The rate of reciprocal translocations in infertile individuals is lower compared to the rate in patients with repeated miscarriage both for males (0.53 % vs 0.86 %) and for females (0.4 % vs 1.54 %).

Three carriers of mosaicism for non centromeric Rea were detected among infertile patients (two cases of N/rcp and one case of N/inv), for a rate of 0.1 ‰, while among patients with repeated miscarriage eight cases were detected (five N/rcp, two N/inv, and one N/del), for a rate of 0.28 ‰. These figures are consistent with the overall lower frequency of carriers of non mosaic chromosomal abnormality among infertile patients of 1.1 % (328/30,376) compared to 1.9 % (505/26,384) among patients with miscarriages.

Reviewing data from prenatal amniocentesis samples, Shapira et al. [22] reported the rates of mosaic balanced reciprocal translocations as <0.02–0.1 per 1,000 samples and suggested that these rates may approximate the true frequency in the general population. However, mosaicism detected in amniocytes might not be confirmed in blood cells postnatally. For example, in the collaborative study of Hsu et al., [23] 13 cases of mosaic balanced reciprocal translocations were identified in 179,663 amniocenteses. However, at birth, five cases were not followed up, five newborns did not have confirmed mosaicism, and in only three cases was mosaicism confirmed. This study also identified four cases of mosaic inversions, with two of the cases confirmed in the newborn infants (0.01 per 1000).

In addition, Hsu et al., [23] commented that when a mosaicism is diagnosed along with a 46,XX cell line, the possibility of maternal cell contamination might be suspected. One more aspect should be taken into consideration, namely that maternal age distribution in couples referred to prenatal testing is different from that in the general population. Further studies are needed before making any conclusion about maternal age effect on formation of mosaicism for structural Reas.

With respect to mosaicism for unbalanced rearrangements, it should be noted that many of the prenatally detected carriers, being abnormal, undergo spontaneous abortion or termination. In the same study of Hsu et al. [23], 17 cases of mosaicism for deletion were detected. Four of them were terminated, three were abnormal at birth, three normal newborns were not followed up, five normal newborns did not have confirmed mosaicism, and two normal newborns had confirmed mosaicism. One of them, with a low-grade mosaicism (2 % of abnormal cells in blood sample) was reported to be normal at 7 months. Of three cases prenatally diagnosed as carriers of mosaicism for ring chromosome, two were abnormal (aborted) and one was a normal infant with a low-grade (8 %) mosaicism.

As noted above, for determination of population rates of mosaicism for structural abnormalities, we chose to analyze combined data from studies of asymptomatic carriers with reproductive failure. Since reproductive failure affects about 15 % of couples, one may calculate population rates of N/rcp, N/inv, N/unbal Rea as 0.02 ‰, 0.005 ‰, and 0.002 ‰, respectively. Therefore, population rates for balanced Reas calculated in the present study, are consistent with figures from prenatal samples [22]. However, it should be noted that these figures are most probably underestimated since in many cases mosaicism goes undetected because of the presence of normal cell line.

It should be stressed that mosaicism confined to the germline is more difficult to detect, and recent evidence suggests that it may be far more widespread than previously assumed [24]. Therefore, we support the view of Shapira et al. [22] and many other researchers: even if mosaicism is not detected, genetic counseling for chromosomally normal parents, with a prior aneusomic offspring or fetal loss, should always address the theoretical possibility of recurrence in a future pregnancy resulting from gonadal mosaicism.

## Conclusions

A high proportion of abnormal Rea cells (>50 %) detected in cultured T-lymphocytes is associated with clinical manifestation of chromosomal imbalance. A strong female prevalence among carriers of mosaicism for unbalanced Rea suggests male-specific selection against abnormal cells rather than impairment of male gametogenesis. The latter suggests a better prognosis for male fetuses. These findings should be taken into consideration when counseling patients referred after a diagnosis of mosaicism for unbalanced rearrangement in a fetus.
